# Radical resection of hepatic alveolar echinococcosis combined with cystic echinococcosis: A case report

**DOI:** 10.1097/MD.0000000000035806

**Published:** 2023-10-27

**Authors:** Xiaoguang Ma, Wei Su, Xiao Wang, Rui Zhao

**Affiliations:** a Department of Hepatobiliary Surgery, Qinghai Red Cross Hospital, Xining, Qinghai, China.

**Keywords:** cystic echinococcosis, hepatic alveolar echinococcosis, radical resection

## Abstract

**Rationale:**

Hepatic Echinococcosis, is a zoonotic Parasitic disease with a worldwide distribution. Clinical cases of alveolar echinococcosis combined with cystic echinococcosis infection are extremely rare.

**Patient concerns:**

A 58-year-old patient had found liver occupying lesions for more than 2 years. A left hepatic alveolar hydatid was found, occupying the entire left half of the liver, with a size of approximately 6.7 cm × 10.9 cm × 8.1 cm. The size of the right liver is about 9 × 8 cm cystic hydatid, mainly located in the S5 segment of the liver. Abdominal examination: the upper abdomen is swollen, and a hard mass can be touched under the right rib margin, with tenderness and no rebound pain. The bowel sounds are normal.

**Diagnoses:**

Abdominal MR shows an increase in liver volume and irregular morphology, with patchy abnormal signal shadows visible in the left lobe of the liver, with a range of approximately 6.7 cm × 10.9 cm × 8.1 cm, low signal on T1WI, low signal on T2WI and FS-T2WI, slightly high signal on diffusion weighted imaging, high signal on apparent diffusion coefficient, no significant enhancement of the lesion after enhancement. In addition, there is a clump like abnormal signal shadow visible in the right lobe of the liver, with a size of approximately 7.9 cm × 7.3 cm × 7.9 cm, low signal on T1WI, mixed high signal on T2WI, high signal on diffusion weighted imaging, mixed signal on apparent diffusion coefficient. Consider: Left lobe alveolar echinococcosis, and right lobe cystic echinococcosis (CE III type).

**Interventions:**

A radical resection was performed, including expanded left hemi-hepatectomy, cholecystectomy, right hepatic lesion resection, partial right hepatic duct resection with right hepatic duct jejunostomy.

**Outcomes:**

The wound healed well after resection. There was no recurrence of TC after 4 years follow-up.

**Lessons:**

The co-infection of alveolar echinococcosis and cystic echinococcosis in a patient is an exceedingly rare occurrence. Radical resection is the only curative treatment.

## 1. Introduction

Hepatic Echinococcosis, is a zoonotic Parasitic disease with a worldwide distribution. Hepatic Echinococcosis can be divided into 2 main types, namely alveolar echinococcosis (AE) and cystic echinococcosis (CE).^[1]^ The final host of AE is mainly foxes and wolves, the Intermediate host is rodents and people, and the final host of CE is mainly dogs, Most of the Intermediate host are sheep, horses, cattle, and humans. The Pathogen transmission of the 2 types of hydatid cysts is the same.^[2]^ Although the incidence rate has decreased year by year, there are still some complicated cases in some areas. Clinical cases of AE combined with CE infection are extremely rare.^[3]^ Our hospital admitted 1 AE combined with CE infection patient, and the report is as follows.

## 2. Case report

The patient, 58 years old, female, was admitted to the hospital on May 10, 2019 due to the main complaint of “liver occupying lesion discovered for 2 years and right upper abdominal pain for 3 days.” The patient has previously been in good health and has lived in the Qinghai region for a long time, in which some areas affected by echinococcosis. Abdominal examination: The sclera is slightly yellow stained, the upper abdomen is swollen, and a hard mass can be touched under the right rib margin, with tenderness and no rebound pain. The bowel sounds are normal. Laboratory examination: Total bilirubin 9.7 umol/L, Alkaline phosphatase 1025 U/L, г- Glutamyl transpeptidase 537 U/L, Albumin 35g/L.

**Figure 1.**
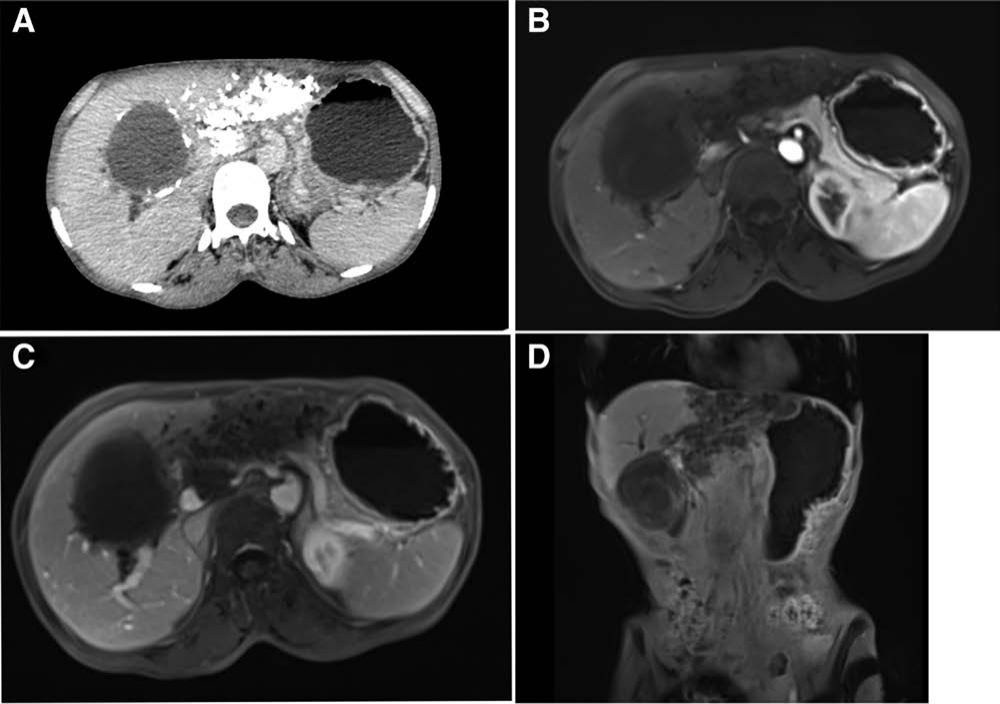
CT and MRI imaging of the lesions: preoperative abdominal CT and MRI of patients showed that there were two lesions of echinococcosis, one in the liver with the size of about 6.7 cm × 10.9 cm in S2–4 segments, and the other with the size of about 7.9 cm × 7.3 cm in S5 segment.

Abdominal contrast-enhanced CT (Fig. 1A) shows normal liver size and morphology, while a circular low-density shadow (CT value = 33 HU) can be seen in the right lobe of the liver without significant enhancement. The edge of the lesion shows linear calcification, with a range of approximately 6.8 × 8.0 cm. Multiple patchy calcifications can be seen in the left lobe of the liver, with the left branch of the portal vein taking shape at the edge of the lesion. The intrahepatic bile duct is slightly dilated, and no enlarged lymph nodes are found in the retroperitoneum or pelvic cavity. Consider CE in the right lobe of the liver (CE II type), AE in the left lobe of the liver, and dilation of the intrahepatic bile duct.

Abdominal MR (Fig. 1B–D) shows an increase in liver volume and irregular morphology, with patchy abnormal signal shadows visible in the left lobe of the liver, with a range of approximately 6.7 cm × 10.9 cm × 8.1 cm, low signal on T1WI, low signal on T2WI and FS-T2WI, slightly high signal on diffusion weighted imaging, high signal on apparent diffusion coefficient, no significant enhancement of the lesion after enhancement, left portal vein, middle hepatic vein, and left hepatic vein not shown. In addition, there is a clump like abnormal signal shadow visible in the right lobe of the liver, with a size of approximately 7.9 cm × 7.3 cm × 7.9 cm, low signal on T1WI, mixed high signal on T2WI, high signal on diffusion weighted imaging, mixed signal on apparent diffusion coefficient, no significant enhancement after enhancement. Consider; Left lobe alveolar echinococcosis, left branch of portal vein, middle hepatic vein, and left hepatic vein invasion; Right lobe cystic echinococcosis (CE III type); Intrahepatic bile duct dilation.

**Figure 2.**
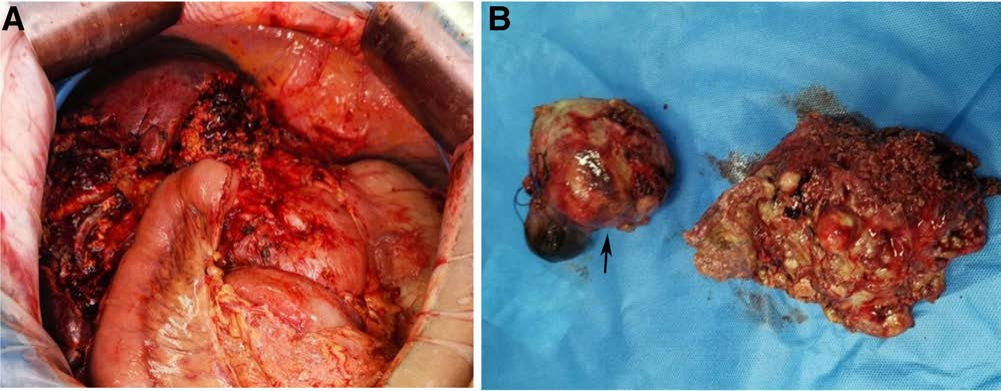
Intraoperative resection and postoperative specimens: (A) expanded left hemi-hepatectomy, cholecystectomy, right hepatic lesion resection, partial right hepatic duct resection and right hepatic duct jejunostomy, (B) two postoperative pathological specimens, is the lesion in S5.

**Figure 3.**
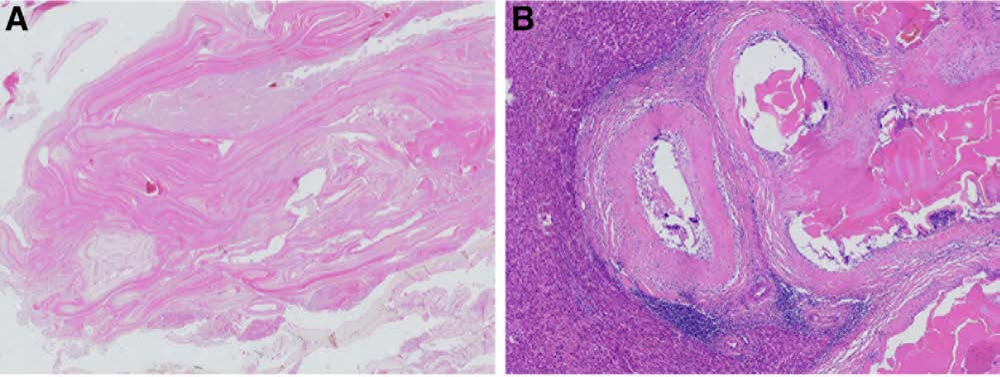
Postoperative pathological sections: (A) cystic echinococcosis in S5 segment (×400), (B) alveolar echinococcosis in S2–4 segments (×400).

Based on the above information, the preoperative diagnosis was “left lobe alveolar echinococcosis and right lobe cystic echinococcosis of the liver”. The preoperative assessment was Child-Pugh grade A, and there were no surgical contraindications for cardiopulmonary function. On May 29, 2019, “expanded left hemi-hepatectomy, cholecystectomy, right hepatic lesion resection, partial right hepatic duct resection and right hepatic duct jejunostomy” was performed. During the surgery, a left hepatic alveolar hydatid was found, occupying the entire left half of the liver, with a size of approximately 12 × 8 cm, invading the left hepatic vein, left branch of the portal vein, left hepatic duct, confluence of left and right hepatic ducts, and some right hepatic ducts. The size of the right liver is about 9 × 8 cm cystic hydatid, mainly located in the S5 segment of the liver. Firstly, an enlarged left hemi-hepatectomy was performed along the right side of the middle hepatic vein, removing the left half liver along with some right hepatic ducts. The right hepatic lesion was removed by intracapsular dissection. Then, the jejunum was severed at a distance of 15 cm from the flexor ligament, and the distal end was lifted, followed by a Roux-en-Y anastomosis of the right hepatic duct jejunum (Fig. 2). 300 mL of intraoperative bleeding was observed without blood transfusion. The first hepatic hilum was blocked 3 times during the operation for a total of 45 minutes. After the surgery, specimens were removed and sent for examination. Pathological examination showed that echinococcosis in the left lobe of the liver was consistent with AE, and echinococcosis in the right lobe of the liver was consistent with CE (Fig. 3). The postoperative diagnosis was consistent with the preoperative diagnosis. After 32 days of hospitalization, the patient recovered and was discharged. Albendazole was taken orally after surgery. No recurrence was observed during 4 years of follow-up.

## 3. Discussion

Multiple or single AE or CE in the liver is common in clinical practice. The clinical cases of AE combined with CE infection are rare and the cause is unknown. As reported by Li TY et al,^[4]^ the causes of 2 types of hydatid disease infection in Qinghai population are animal husbandry, traditional nomadic production mode, overrunning of wild dogs, drinking unclean surface water, and bad hygiene habits such as no or very little hand washing. It is suggested that the epidemic of human echinococcosis in this region is caused by a combination of multiple factors. Another epidemiological feature of hydatid disease is that it is geographically distributed,^[1]^ and the species and types of hydatid disease are different according to the different living areas. For example, in southern Qinghai Province, Chengduo County belongs to the high incidence area of AE, and Nangqian County belongs to the high incidence area of CE.^[4]^ Some studies have also reported that the type of hydatid parasites is related to the difference of their final hosts.^[5]^ At present, there is no literature and research report on the reason for the regional distribution of hydatid disease. Based on the clinical and epidemiological characteristics, we hypothesize that the patient was infected with AE in the endemic area of alveolar echinococcosis and then infected with CE in the endemic area of cystic echinococcosis. Further studies are needed to confirm the exact reason for the co-infection of the 2 types of echinococcosis.

The preferred treatment for hepatic echinococcosis is still radical surgical resection, such as complete external cystectomy, anatomical hepatectomy (AR), ex vivo liver resection, liver transplantation and other surgical methods.^[6]^ For CE, Wen H et al^[7]^ further proposed an improved method of radical resection based on the external cyst resection of hepatic hydatid cyst, namely complete removal of the cyst close to the external cyst wall of hydatid cyst or removal of the cyst after decompression, which was called complete removal of the cyst of hepatic cystic echinococcosis. This operation not only better solves the 2 major problems of postoperative recurrence and biliary fistula. It also solves the problem that patients with multiple hepatic CE, cirrhosis and limited liver volume retain more liver volume and reduce the risk of liver failure. With the deepening of the concept of precise hepatectomy, anatomical hepatectomy has been gradually applied to the treatment of AE, but whether this surgical method can reduce the recurrence rate of patients is still inconclusive.^[8]^ Therefore, the specific surgical method for hepatic alveolar echinococcosis still needs to be further clarified by RCT studies with large samples. In our center, laparoscopic or open radical resection is adopted for early AE, and ex vivo liver resection + autologous liver transplantation is the first choice for the treatment of end-stage AE, which has the advantages of no need to find a liver source, no need for immunosuppressive therapy, and no increase in the incidence of postoperative complications, thus becoming an ideal surgical procedure for end-stage AE.

We followed the principle of radical resection and adopted the surgical strategy of complete extracapsular resection and anatomical extended left hemi-hepatectomy. The patient recovered well and no recurrence was found after 4 years of follow-up. However, this is only a single case. For this kind of patients with AE and CE infection, the cause of the occurrence, the advantages of surgical methods, and the dosage of postoperative drugs have not yet been determined, and more cases of multi-center studies are needed to make an objective evaluation.

## 4. Conclusion

The co-infection of alveolar echinococcosis and cystic echinococcosis in a patient is an exceedingly rare occurrence. For patients with complex echinococcosis, there was no recurrence after 4 years of follow-up, which indicates that radical resection is an effective cure.

## Author contributions

**Data curation:** Xiao Wang.

**Investigation:** Wei Su.

**Writing – original draft:** Xiaoguang Ma.

**Writing – review & editing:** Rui Zhao.
